# Lightweight Vision Transformer for Frame-Level Ergonomic Posture Classification in Industrial Workflows

**DOI:** 10.3390/s25154750

**Published:** 2025-08-01

**Authors:** Luca Cruciata, Salvatore Contino, Marianna Ciccarelli, Roberto Pirrone, Leonardo Mostarda, Alessandra Papetti, Marco Piangerelli

**Affiliations:** 1 Department of Engineering, University of Palermo, 90128 Palermo, Italy; luca.cruciata@unipa.it (L.C.); salvatore.contino01@unipa.it (S.C.); roberto.pirrone@unipa.it (R.P.); 2 Department of Industrial Engineering and Mathematical Sciences, Poytechnic University of Marche, Via Brecce Bianche 12, 60131 Ancona, Italy; m.ciccarelli@staff.univpm.it (M.C.); a.papetti@staff.univpm.it (A.P.); 3 Department of Mathematics and Informatics, University of Perugia, Via Vanvitelli 1, 06123 Perugia, Italy; leonardo.mostarda@unipg.it; 4 Computer Science Division, School of Science and Technology, University of Camerino, Via Madonna delle Carceri 7, 62032 Camerino, Italy; 5 Vici & C. S.p.A., Via Gutenberg 5, 47822 Santarcangelo di Romagna, Italy

**Keywords:** computer vision, deep learning, ergonomic risks, human-centered manufacturing, posture recognition, work-related musculoskeletal disorders, attention mechanism

## Abstract

Work-related musculoskeletal disorders (WMSDs) are a leading concern in industrial ergonomics, often stemming from sustained non-neutral postures and repetitive tasks. This paper presents a vision-based framework for real-time, frame-level ergonomic risk classification using a lightweight Vision Transformer (ViT). The proposed system operates directly on raw RGB images without requiring skeleton reconstruction, joint angle estimation, or image segmentation. A single ViT model simultaneously classifies eight anatomical regions, enabling efficient multi-label posture assessment. Training is supervised using a multimodal dataset acquired from synchronized RGB video and full-body inertial motion capture, with ergonomic risk labels derived from RULA scores computed on joint kinematics. The system is validated on realistic, simulated industrial tasks that include common challenges such as occlusion and posture variability. Experimental results show that the ViT model achieves state-of-the-art performance, with F1-scores exceeding 0.99 and AUC values above 0.996 across all regions. Compared to previous CNN-based system, the proposed model improves classification accuracy and generalizability while reducing complexity and enabling real-time inference on edge devices. These findings demonstrate the model’s potential for unobtrusive, scalable ergonomic risk monitoring in real-world manufacturing environments.

## 1. Introduction

Work-related musculoskeletal disorders (WMSDs) remain among the most pressing occupational health challenges worldwide, particularly in manual and industrial labor contexts. In the EU alone, WMSDs affect nearly 60% of workers, regardless of the sector or job role [1]. These disorders originate from a complex interplay of biomechanical, organizational, and psychosocial risk factors and commonly manifest in the back, shoulders, neck, and upper limbs. Among the key physical risk factors, manual material handling, awkward postures, repetitive motions, and exposure to vibrations have been consistently associated with the onset and progression of musculoskeletal disorders. Although limited exposure to these factors may not cause immediate harm, sustained or high-intensity demands often exceed physiological tolerance, increasing the risk of injury over time [2]. Large-scale surveys highlight the scale of this problem as follows: in Europe, 44% of employees are exposed to tiring or painful postures for at least a quarter of their working time, with 13% reporting such exposure for more than three-quarters of their shift. Likewise, repetitive movements of the hands or arms affect 61% of workers, with a significant share enduring these conditions for most of their workday [2]. Beyond pain and disability, WMSDs also involve high economic costs in terms of reduced productivity, absenteeism, and health care burdens [3]. The manufacturing sector, in particular, suffers from high rates of sick leave and absenteeism due to WMSDs, with the back and upper limbs among the areas most frequently affected [4]. For example, in Germany alone, WMSDs account for an estimated EUR 6.45 million in annual production losses and EUR 10.63 million in lost gross value added [5].

This scenario highlights the urgent need for effective risk assessment strategies capable of identifying hazardous conditions before they result in injury. To this end, traditional ergonomic assessment methods—such as OCRA (OCcupational Repetitive Action), REBA (Rapid Entire Body Assessment), and RULA (Rapid Upper Limb Assessment)—have been widely adopted due to their ease of use and standardization. These methods involve scoring postures and loads based on visual inspections or video analysis by trained evaluators. However, they suffer from limitations, including evaluator subjectivity, limited temporal resolution, and a strong reliance on manual interpretation, making them impractical for continuous, real-time, or large-scale monitoring [6,7]. Finally, while detailed biomechanical simulations offer insights into physical load distributions under postural strain, these approaches are computationally intensive and not directly applicable to scalable in-field ergonomic assessment [8].

These constraints have motivated the development of automated, objective methods based on sensor technologies and computer vision. Motion capture has become a cornerstone of biomechanical research, offering reliable quantitative insights into human movement dynamics [9]. A wide range of technologies are used for this purpose, including optical, wearable, and hybrid systems [10]. In laboratory settings, marker-based optical motion capture systems equipped with infrared cameras are still regarded as a gold standard due to their precision. However, their applicability is largely restricted to controlled environments, and they involve high setup costs and complex data processing workflows. Among more deployable alternatives, inertial measurement units (IMUs) have received widespread attention due to their portability and ability to capture full-body kinematics in non-laboratory environments. IMUs typically integrate accelerometers, gyroscopes, and magnetometers, enabling joint angle computation and motion segmentation. These systems have been applied to diverse ergonomic scenarios, such as warehouse operations [11], muscle load monitoring via EMG [12], and agricultural harvesting tasks [13]. A modular low-cost motion capture system capable of estimating full-body segment orientations using quaternion-based Kalman filtering, with additional gait recognition functionality, was proposed in [14]. More recently, an open source, sensor-based pipeline combining custom IMUs and OpenSim inverse kinematics, has been introduced and validated in a real automotive plant with <5% deviation in RULA scores compared to commercial references [15]. Despite their demonstrated potential, the use of IMUs in ergonomic risk assessment remains limited by several practical constraints [16]. These include sensor drift, the need for recalibration in dynamic conditions [17], discomfort from prolonged wear [18], short battery life [19], and the specialized expertise required for data elaboration and interpretation [20]. Moreover, some authors emphasized that the effective integration of wearable motion capture into ergonomic workflows often entails additional analytical effort, which can reduce operational efficiency and hinder widespread adoption [21]. Economic factors also play a role, with high-end systems remaining unaffordable for many small and medium enterprises [22]. Still, recent studies point to measurable improvements in ergonomic conditions through wearable technologies, with artificial intelligence emerging as a key enabler for automated risk detection [23]. Despite their growing technical maturity, the integration of wearable systems into routine industrial workflows for ergonomic risk monitoring remains limited, primarily due to usability constraints and the complexity of post-processing pipelines. While the development of tools that automate post-processing and support risk factor identification can help address many of the current limitations of sensor-based systems, their use in real-time applications—such as delivering immediate feedback to workers or managing human–machine collaboration—remains constrained. In such contexts, vision-based systems play a complementary role, enabling more responsive and comprehensive ergonomic monitoring.

As a result, interest has grown in vision-based markerless motion capture systems that leverage computer vision and machine learning (ML) or deep learning (DL) to estimate postures from RGB or RGB-D video [24,25]. These systems offer the advantage of being fully non-invasive, low-cost, and scalable. These approaches typically follow a two-stage pipeline, outlined as follows: first, body pose is estimated from RGB or RGB-D video using ML DL methods; then, joint angles or geometric features are extracted and mapped to ergonomic risk indices such as RULA, REBA, or OWAS. Technically, vision-based systems can be classified by spatial representation (2D vs. 3D), input modality (monocular or multi-view), and model architecture (single- or multi-person pipelines). Early implementations used 2D detection with OpenCV and Haarcascade [26], while more sophisticated models such as OpenPose [27] and VoxelPose [28] enabled 3D pose estimation via multi-camera or volumetric strategies. However, challenges such as occlusion, variability in lighting, camera calibration, and restricted field of view still compromise their performance in industrial settings [29,30,31,32].

Together, these points motivate the present study, which combines a single-view pose-estimation backbone with a lightweight temporal graph encoder. A wide range of studies have applied CNN-based methods for pose estimation and activity recognition. One approach employed a 3D CNN to classify writing postures [33], while other works proposed temporal CNNs to extract spatiotemporal features for human action recognition [34,35]. Several systems estimate joint angles from skeletons to compute ergonomic scores. For instance, RULA scores have been predicted using 2D joint data combined with Euclidean and angular metrics [36].

Other similar efforts rely on skeleton-based representations with varying levels of accuracy [24,37,38,39]. Despite promising results, these methods often lack robustness in uncontrolled industrial environments and remain sensitive to lighting, occlusion, and camera viewpoint. Furthermore, they usually depend on intermediate steps (e.g., joint angle estimation) that introduce error propagation and reduce real-time feasibility.

Recent advances have introduced annotated datasets and benchmarking efforts to improve vision-based ergonomic scoring. For instance, some authors extended the COCO dataset with ergonomic key points for REBA assessment and developed REBAPose, which improved pose inference and scoring accuracy [27]. However, this work remains confined to 2D and lacks validation in operational scenarios. PoseChecker [40] represents a validated markerless motion capture tool that aligns closely with marker-based optical motion capture and IMU references in terms of joint tracking and ergonomic scoring accuracy. Related to this, vision-language models have also been proposed to sanitize industrial datasets containing noisy or weak labels. For instance, some authors proposed VLSR, a CLIP-based framework that aligns image and text embeddings to cluster, refine, and consolidate labels in multi-label manufacturing datasets. While our study relies on high-fidelity motion capture for ground truth, such techniques are highly relevant for enabling scalable data annotation in real-world applications [41]. However, results may not generalize to real-world industrial scenarios characterized by occlusions and variable lighting. A 2D RGB MoCap system was evaluated across multiple real-world industrial environments, confirming its practical potential, although performance varied under conditions involving occlusions and nonstandard layouts [42]. Additional comparisons between Kinect V2, OpenCV, and IMU-based systems for torso posture estimation indicated that Kinect remains a viable option, despite its sensitivity to lighting conditions and limited tracking scope [43]. In addition, hybrid frameworks are emerging that combine posture detection with ergonomic reasoning or decision making. As an example, the integration of VideoPose3D with fuzzy logic for RULA scoring and task reassignment to collaborative robots has shown promising results in productivity and ergonomic load reduction, although it is limited to single-user offline setups [44].

An early work demonstrated that even a compact CNN trained on roughly one thousand images could already classify four industrial lifting postures with ≈94% accuracy, opening the door to camera-based ergonomic screening [45]. Building on that proof of concept, a Mask-R-CNN person segmentation step was added in [46]. This way posture and fall detection remained reliable in cluttered domestic rooms at real-time speed (≈28 fps on a GTX-1080). To cope with short-term occlusions common on factory floors, one study fused four consecutive frames within a multi-stream CNN, boosting the F-score to 0.88 even when a worker was momentarily hidden by a workpiece [47].

Although these vision-only systems rely on RGB or depth imagery, several groups began to exploit estimated 3-D skeletons for finer-grained reasoning. Çalışkan (2022) [48] tiled entire joint-coordinate sequences into “posture mosaics”, achieving perfect accuracy on the small KARD and MSR-Pairs datasets—an impressive result. Seeking a direct link to ergonomic scores, Hossain et al. (2023) [49] trained on 3.6 million synthetic poses to map 3-D key-points straight to REBA risk levels, achieving 89% overall accuracy but still missing around 29% of minority high-risk cases.

A complementary direction was explored by Kwon et al. (2022) [50], who showed that monocular RGB cameras could approximate expert RULA ratings (ρ=0.82) on their DyWHSE dataset—although heavy self-occlusion and personal protective equipment remained problematic. Finally, Jung et al. (2025) [51] reminded the community that accurate posture labels are only a means to an end; using 7270 compensation records, they predicted the work-relatedness of shoulder disorders with 84% accuracy, demonstrating how machine learning can close the loop between exposure assessment and clinical outcomes.

The following three unresolved challenges emerge from this body of work:

- Occlusion resilience—despite temporal fusion and person segmentation, long or permanent occlusions still degrade joint tracking and risk estimates.
- Dataset realism and scale—many claims of near-perfect accuracy rely on small or proprietary datasets that do not capture the lighting, attire and workflow variability of real factories.
- Outcome linkage—only a handful of studies quantify posture recognition through indices directly linked to musculoskeletal risk prediction.

In general, most vision-based systems rely on skeleton reconstruction and joint angle estimation to derive ergonomic scores, which may not be optimal under industrial constraints. In this context, the present study contributes to the field by introducing the use of a state-of-the-art neural network, the Vision Transformer (ViT). Compared to convolutional network approaches, this network manages more efficiently processing of the relevant information, without requiring extensive pre-processing of important image features. Furthermore, the ViT used in this work manages to provide the classification for each of the body parts through a single training, in contrast to the first version of this approach [52]. In this way, the overall computational cost is reduced considerably by speeding up the training and inference phases; the limited number of parameters could favour near real-time applications of the prediction, returning predictions in real time with a very high accuracy. The main contributions are summarized as follows:

- A vision-based ergonomic risk classification framework is proposed, leveraging a ViT architecture for direct analysis of RGB images, without relying on skeleton reconstruction or joint angle estimation.
- The system is designed to classify multiple body regions simultaneously within a single model, reducing computational complexity and simplifying training and inference procedures.
- The approach is validated on a multimodal dataset of simulated industrial tasks, reflecting realistic working conditions and addressing common challenges such as posture variability and occlusion.
- The classification output is directly linked to ergonomic risk indices, enabling frame-level posture risk assessment applicable to industrial monitoring scenarios.

This paper is organized as follows. Section 2 describes the data acquisition protocol, the dataset processing pipeline, and the architecture of the ViT-based classifier. Section 3 presents the experimental setup, evaluation metrics, and classification results. Section 4 discusses the findings, highlighting the strengths and limitations of the approach. Finally, Section 5 concludes the paper and outlines possible future research directions.

## 2. Materials and Methods

### 2.1. Dataset Creation

The dataset used in this study was originally developed and presented in our previous work, SPECTRE, where we proposed a preliminary vision-based method for ergonomic risk assessment in collaborative industrial environments [52]. The data acquisition protocol, fully described in that paper, involved the simulation of typical manual operations performed in an Italian kitchen manufacturer, such as handling, assembly, and quality control, reproduced in a laboratory setting. The acquisition setup was designed to replicate realistic industrial conditions, including poor lighting, obstacles, and dynamic operator movements, thereby introducing natural variability in postures, occlusions, and viewpoints.

Data acquisition was carried out using a dual system composed of a full-body inertial motion capture system (Xsens MVN) and a high-resolution RGB video camera, both operating at 60 frames per second. The motion capture system comprised 17 wireless MTw sensors positioned according to standard biomechanical placement guidelines, allowing the continuous recording of joint angles and body segment orientations. The video stream was used as input for the image-based posture classification, while the kinematic data served as ground truth for ergonomic labeling. Synchronization between the two data streams was ensured by displaying the motion capture frame counter within the camera’s field of view at the beginning of each recording. This enabled precise alignment of each video frame with the corresponding row in the Xsens output.

**Figure 1:**
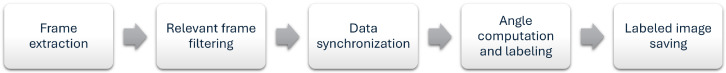
Dataset generation pipeline.

For each frame, the motion capture data were used to compute joint angles and segment orientations for the following key body parts: upper and lower arms, wrists, neck, and trunk, separately for left and right sides. A custom algorithm processed the motion capture output to derive these joint angles and compare them against threshold values derived from the RULA method [53]. Each body segment in each frame was then labeled as either “OK” (ergonomic posture) or “KO” (non-ergonomic posture), depending on whether the angles exceeded the critical values defined by RULA for that joint [52]. Dataset generation was performed through an automated pipeline with the following steps (Figure 1):

1. Extraction of individual frames from the video recording.
2. Removal of non-relevant frames (e.g., neutral postures, transitions).
3. Matching of each frame to its corresponding motion data entry.
4. Calculation of joint angles and ergonomic risk label for each body segment.
5. Saving each frame as a labeled image, where the filename encodes the frame number and the ergonomic classification for the specific body part.

**Figure 2:**
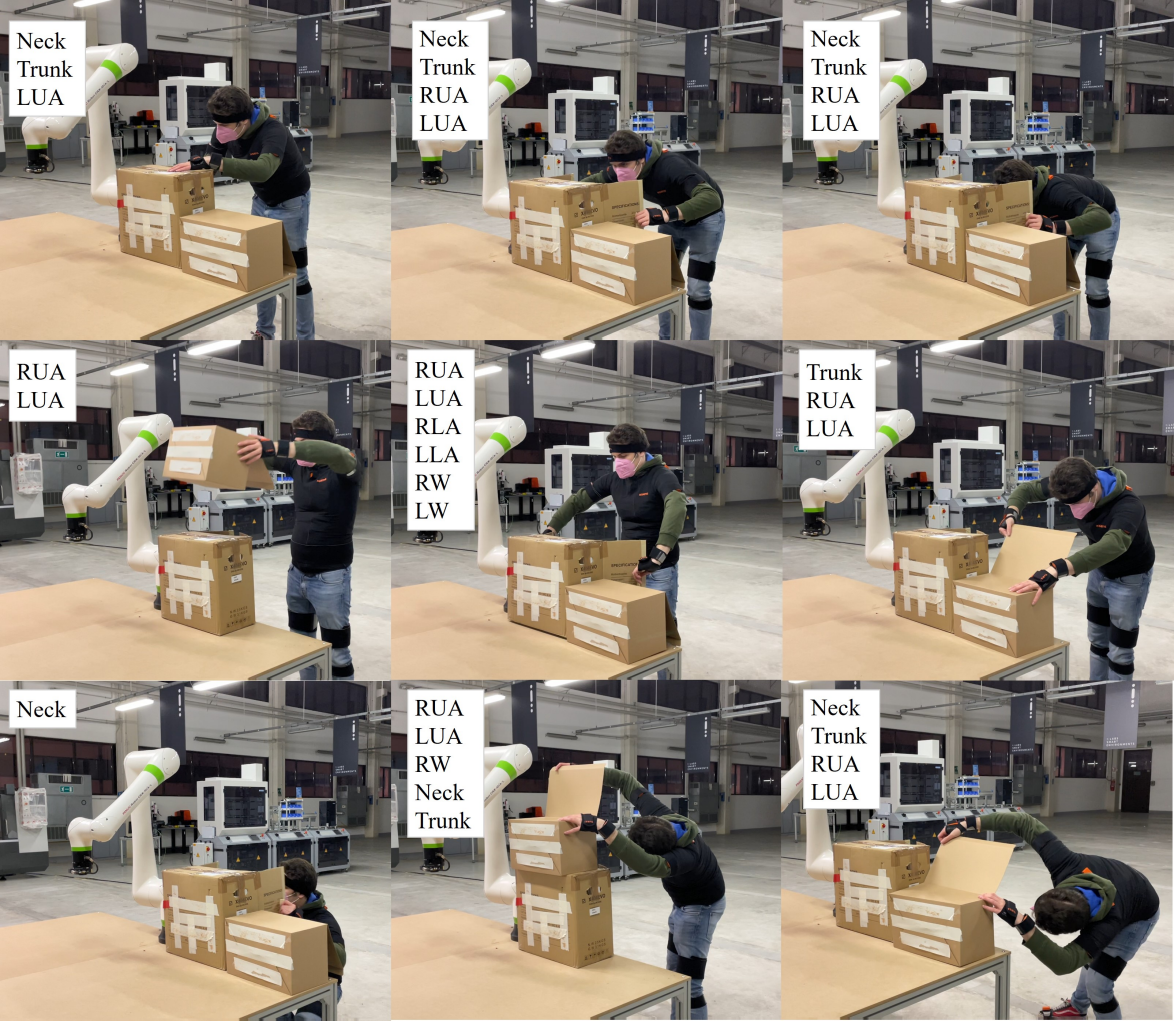
Example frames extracted from the acquired dataset. The labels indicate the body segments identified as risky in each posture.

An illustrative example of the acquired data and the corresponding ergonomic risk labels is shown in Figure 2.

### 2.2. Data Processing

**Figure 3:**
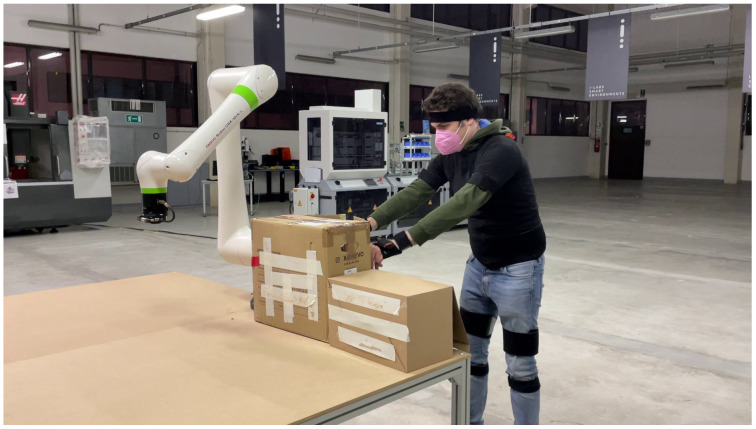
Example image from the dataset used during training and validation phases (Adapted from [52]). The features extracted from this image are used for human pose classification in real-world factory environments.

The dataset used consists of 47,822 samples with an active/inactive ratio of ∼6% for each of the classes present. The labels reported as *active* will describe the anatomical regions that have an incorrect anatomical conformation, while *inactive* will code the anatomical regions that have a correct and physiological position. In contrast to the previous work, the pre-processing of the images did not involve the use of MEDIAPIPE (https://github.com/google-ai-edge/mediapipe, accessed on 9 May 2025) to crop the subjects from the global image. This design choice has been made in order to build a computationally cheap pipeline, thus speeding up the inference process. In fact, leaving the image raw (as shown in Figure 3) allows inference to be performed using the frame acquired straight from the camera, which increases the amount of information available from it and complicates the classification process. To validate the classification performance in a more robust and reliable way, a 5-fold cross-validation has been applied, splitting the dataset into 5 different folds and evaluating the average performance on each generated validation set. This approach allows for more stress in the training phases, ensuring greater reliability on the results obtained, displayed as the average performance on each of the folds.

### 2.3. Network Architecture

**Figure 4:**
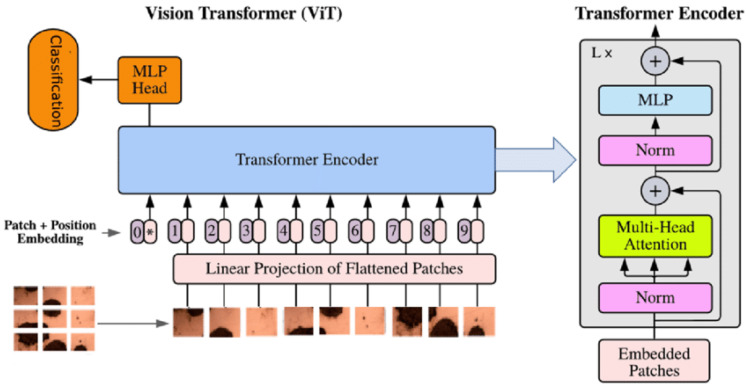
The ViT architecture employed in the pose classification pipeline. The input image is split into patches, which are embedded and passed through a series of transformation layers to produce a classification output.

The architecture used is a lightweight variant of ViT [54], designed to reduce the number of parameters while keeping classification performance high. This family of architectures, as is well known, are able to extract both local and global information, obtaining contextualized information that overcomes the limitations of CNNs. The latter, prior to the advent of Transformers, dominated the field of computer vision, finding applications in all domains of study, despite limitations related to the inability to abstract local information within complex contexts. For this reason, ViTs with their self-attention mechanisms have been able to improve global classification and segmentation performance by being able to correlate long-distance information within richly detailed images. One of the main characteristics of ViTs is related to the patching mechanism of the input image. In fact, an image whose dimensions are typically represented by H×W×C is divided into square paths of dimension P×P. Each patch is linearized into a vector of dimension $P^{2}\times C$ and subsequently encoded in a latent space through a Multilayer Perceptron (MLP). The result obtained is a sequence of vectors $\left\{x_{1},x_{2},\ldots ,x_{n}\right\}$, where $n=\frac{HW}{P^{2}}$. This linearization process leads to a loss of spatial information, which is compensated for by the addition of positional embedding to keep it in memory. Each layer of the ViT typically consists of a Multi-Head Self-Attention (MHSA) module, a feed-forward (MLP) layer, a normalization layer (LayerNorm), and residual blocks to propagate lossless information forward. Classification is achieved through a token [CLS] that will be used as input for the classification block. The general schematization of the operation of a ViT is shown in Figure 4.

In summary, the main difference between CNNs and ViTs lies in the feature extraction mechanism;l the former use locally static convolutional filters, whereas ViTs adopt a global attention mechanism that can dynamically adapt to the relative importance of the various image regions. In the proposed work, a ViT has been implemented that will be described in detail in the next section, which aims to surpass in terms of efficiency and performance classical convolutional architectures such as those used for the same task in the previously published work [52].

#### Architecture Parameters

**Figure 5:**
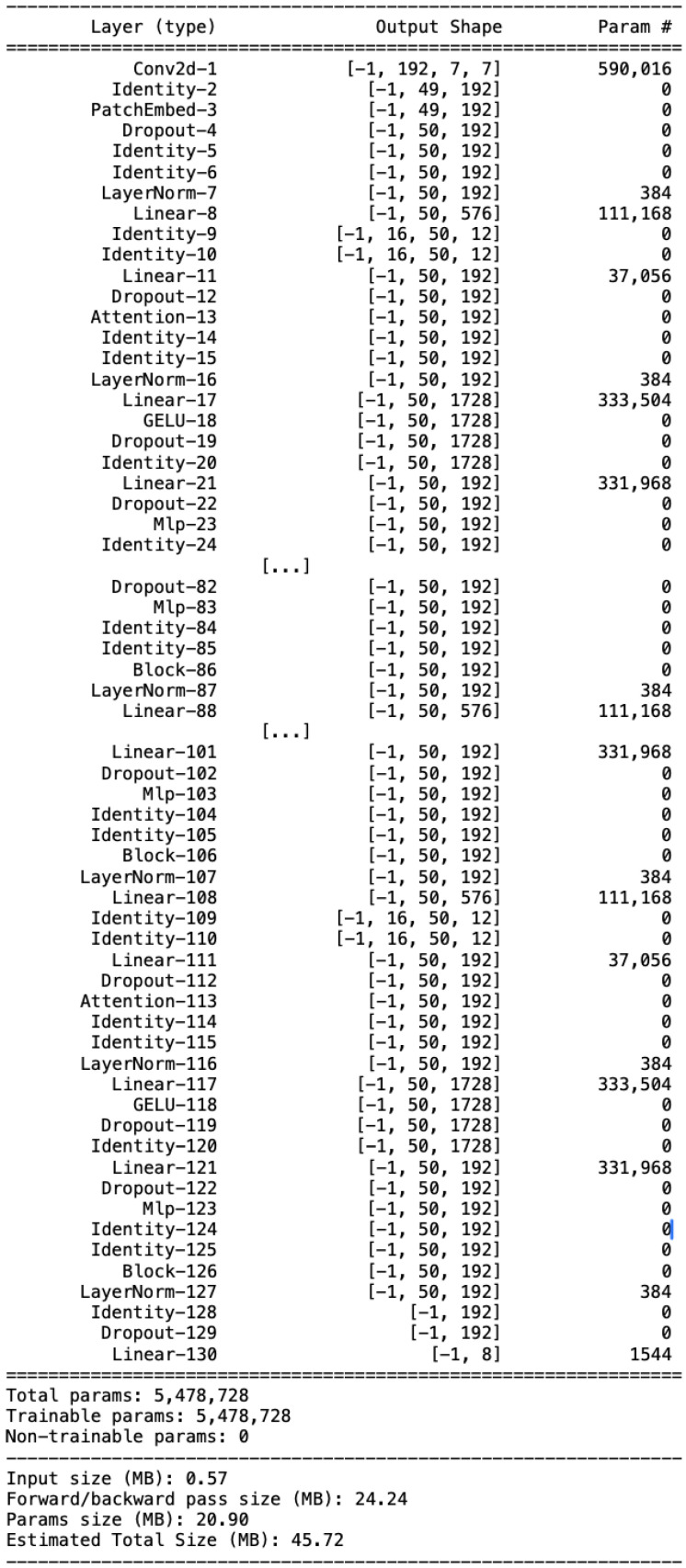
Schematic summary of the architecture used. This representation shows all the layers and trainable parameters for each of them.

To evaluate the impact of hyperparameters on performance, a search grid was used, with the values shown in Table 1. The configuration chosen for the proposed architecture specifically includes the use of a patch_size parameter of 32. This choice allows us to subdivide the starting image into 49 patches of size 32×32, thus providing the right trade-off between local and global information. In fact, given the nature of the image, it is essential to be able to extrapolate the features relating to the subject to be classified. In contrast to the previous approach, as discussed in Section 2.2, the proposed model does not use the cropped images but uses the entire frame. In order to improve the computational efficiency, different patch embedding values have been tested starting from 1024 (32×32), using all the information in the patches. The ideal size was identified as patch_embedding = 192, which allowed the efficient integration of the information present in each patch. To maximize feature extraction capabilities, six transformer blocks were inserted, each with 16 heads for Multi-Head Attention. Finally, self-attention bias has been added to extend the learning capabilities by allowing the architecture to better adapt to the data. Despite the depth, the neural architecture had a small number of parameters (around ∼ 5M) as shown in Figure 5.

**Figure 6:**
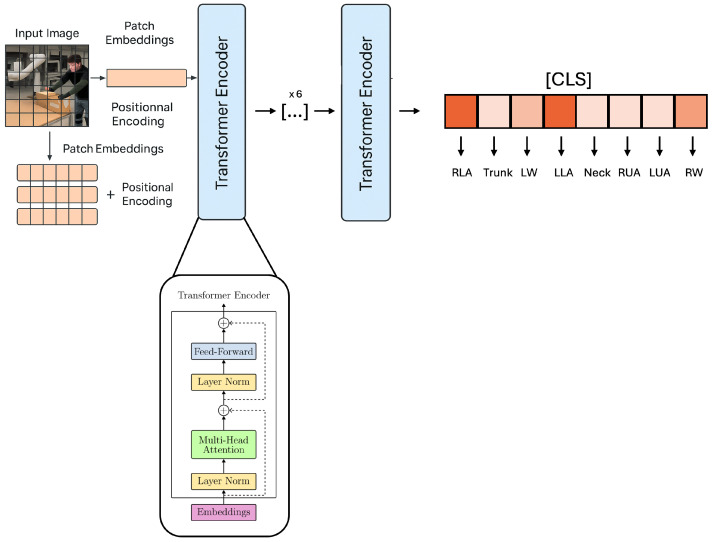
Visual representation of the ViT architecture proposed and employed in this work. The first part of the architecture shows the mechanism for generating patches and positional embeddings that will become inputs for the first transformer encoder. On the right side of the architecture, we see the CLS token that will lead to the prediction for each of the classes.

In summary, the best architecture employed receives as input images of size 224×224, which will be divided into patches of size 32. This will form the input representation for the 6 consecutive transformer blocks each of which will consist of a Multi-Head Attention with a number of heads equal to 16. A CLS token (Class token) used during the training phases will be added to each image initially to associate the analyzed patches with the reference class. This CLS token will be used in the output to the architecture to return the prediction. A visual representation of the final architecture is shown in Figure 6.

## 3. Results

The following section shows the results obtained with the architecture proposed in the present paper. The validation of our work has been carried out using the following metrics:

- Accuracy, i.e., the percentage of correct predictions out of the total number of predictions
  $$\mathrm{Accuracy}=\frac{\mathrm{TP}+\mathrm{TN}}{\mathrm{TP}+\mathrm{TN}+\mathrm{FP}+\mathrm{FN}}$$
  where TP represents the True Positives, TN represents the True Negatives, FP represents the False Positives, and FN represents the False Negatives.
- Area Under the Curve (AUC) reporting the model’s ability to discriminate between the two classes
  $$\mathrm{AUC}=\int_{0}^{1}\mathrm{TPR}\left({\mathrm{FPR}}^{-1}\left(x\right)\right)\;dx$$
  where TPR is the True Positive Rate and FPR is the False Positive Rate.
- Precision that considers among all positive predictions those that are truly positive
  $$\mathrm{Precision}=\frac{\mathrm{TP}}{\mathrm{TP}+\mathrm{FP}}$$
- Recall (Sensitivity) which measures the TP predictions that have been detected, giving a measure descriptive of the discriminative capacity of the model
  $$\mathrm{Recall}=\frac{\mathrm{TP}}{\mathrm{TP}+\mathrm{FN}}$$
- F1-Score, i.e., the harmonic mean between Precision and Recall, which provides an analytical compromise for highly unbalanced datasets such as the one under study
  $$F_{1}=2\cdot \frac{\mathrm{Precision}\cdot \mathrm{Recall}}{\mathrm{Precision}+\mathrm{Recall}}$$

In order to facilitate the structure of the results shown, the explicit name of the labelled classes has been coded as shown in Table 2.

Table 3 shows the average results obtained with the 5-fold cross-validation with reference to the metrics described above.

**Figure 7:**
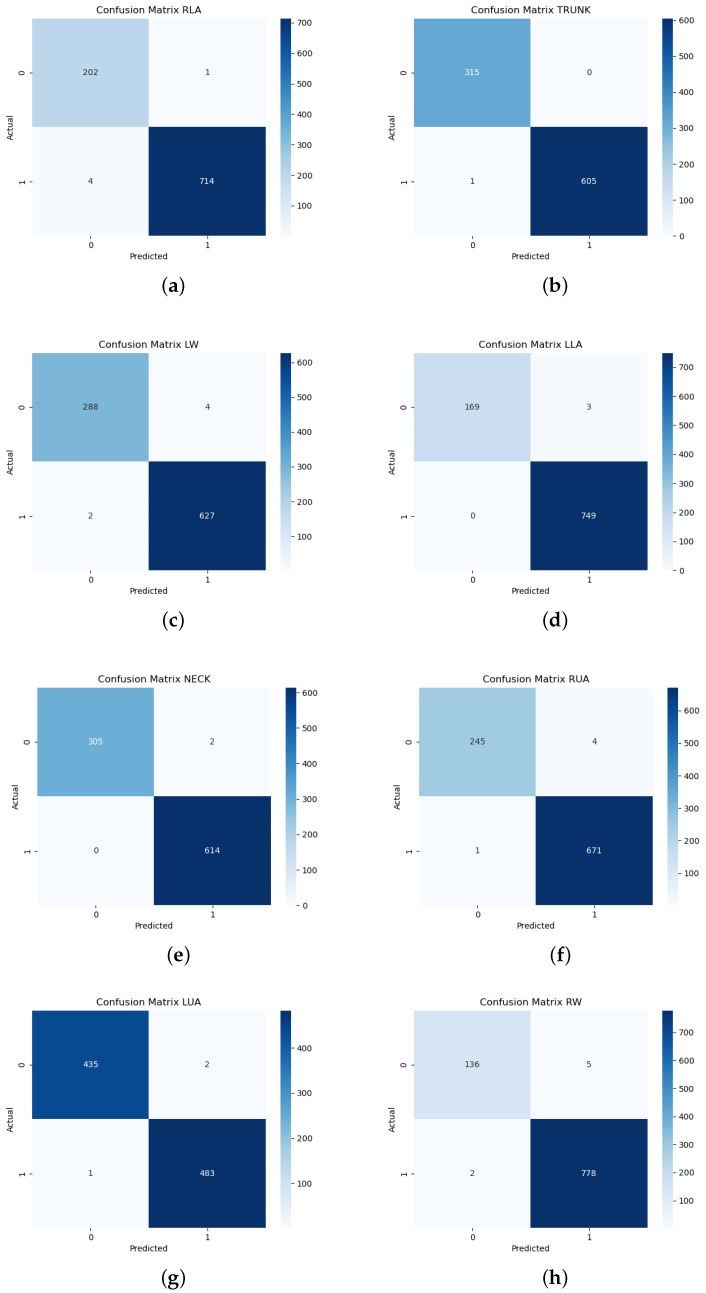
(**a**–**h**) Confusion Matrices for SPECTRE-ViT.

**Figure 8:**
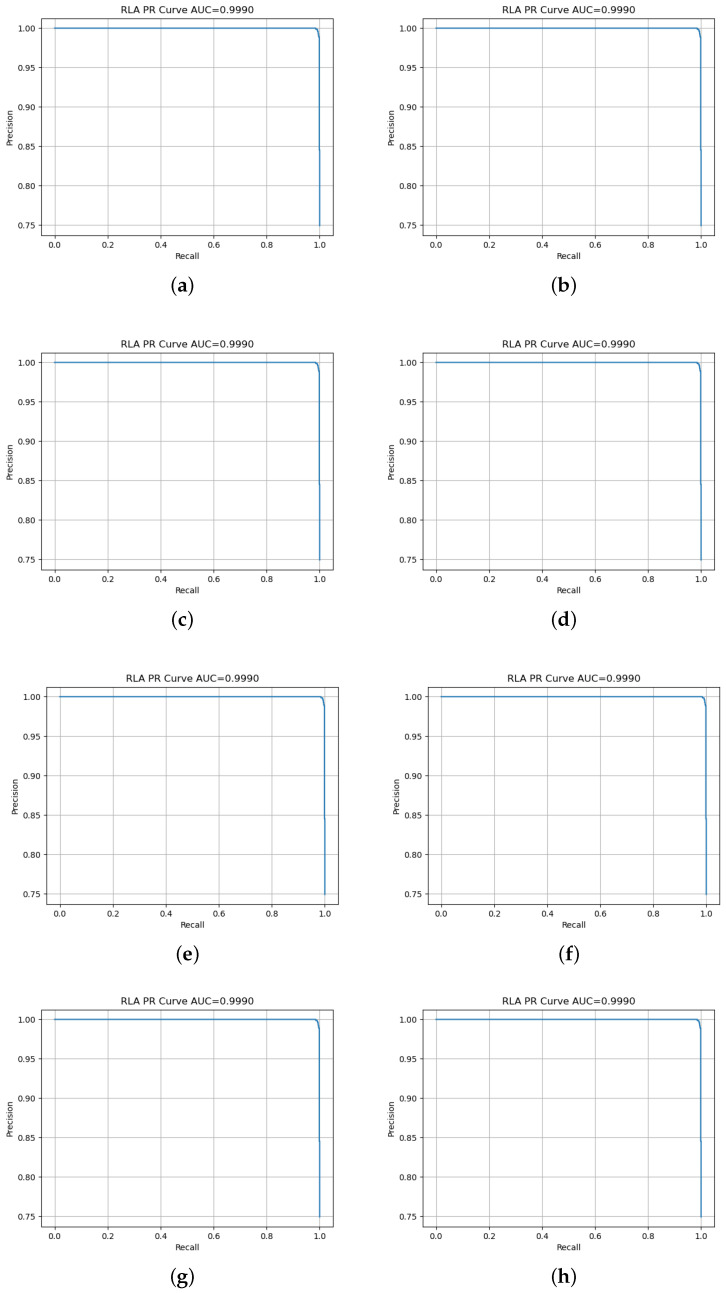
(**a**–**h**) PR Curves for SPECTRE-ViT.

As can be seen, the results obtained are excellent for each classes. These results remain consistent considering the use of K-Fold cross-validation, further demonstrating the reliability of our architecture. Further proof of the outstanding performance is provided by the confusion matrices shown in Figure 7a–h. As can be observed, the confusion matrices show the very high performance of the model on all the classes analyzed. As can be observed, the amount of prediction of FP and FN is negligible or even absent for the classes TRUNK, LLA and NECK. Further evidence is provided by the Precision–Recall curves, as can be seen in Figure 8a–h. These curves show consistency and uniformity between the different classes, with a constant AUC value of 0.9990. This data support the confusion matrix, underlining a very favorable balance between precision and recall. The curves tend to maintain precision close to unity even for very high recall values, showing that the model is able to identify almost all positives without introducing significant false positives.

As can be seen for all classes, the error does not exceed 4 misclassifications, with a peak of 5 misclassifications for the Right Wrist class (RW). This occurrence could be caused by the pose and perspective of the acquired image. In fact, as can be seen in Figure 3, the right wrist is often covered by the equipment that the operator is handling. Despite this additional challenge, the proposed architecture succeeds in extracting the features to correctly discriminate the current pose that the anatomical region of reference is taking in that particular frame thanks to the lightweight ViT’s abilities to extract information. In fact, this architecture, thanks to the global/local mechanisms, manages to integrate the features for the correct class using contextual information, thus making up for the lack of information for hidden classes (e.g., RW).

In addition, a quantitative comparison in terms of Precision–Recall curves (shown in Table 4 has been conducted with the previous SPECTRE approach, highlighting numerically how the use of SPECTRE-ViT improves prediction accuracy while significantly reducing computation time.

## 4. Discussion

The results obtained in this study confirm the effectiveness of a ViT-based approach for frame-level ergonomic posture classification in an industrial context. The system demonstrated remarkably high performance across all anatomical regions evaluated, achieving an average AUC above 0.996 and F1-scores exceeding 0.99 for most classes, as summarized in Table 3. These findings underscore the robustness and reliability of the proposed architecture, particularly when benchmarked against existing state-of-the-art approaches in ergonomic risk estimation.

The proposed ViT-based architecture also offers clear advantages over existing vision-based posture recognition systems. Conventional approaches often rely on skeleton estimation or intermediate joint-angle computations, typically extracted via tools such as OpenPose or Kinect. These intermediate steps introduce inherent limitations, including error propagation, occlusion sensitivity, and viewpoint dependency. Moreover, many vision-based systems are validated on constrained or small-scale datasets, leading to inflated performance metrics that may not generalize to realistic industrial environments.

In contrast, our approach directly classifies ergonomic posture from raw RGB frames, avoiding the skeleton reconstruction pipeline entirely. This not only streamlines the processing workflow but also results in significant performance improvements. As shown in Table 3 and Table 4, our method outperforms the CNN-based SPECTRE framework across all evaluated classes, with AUC margins reaching up to 12.9% for challenging regions such as the Right Wrist (RW), which is typically subject to frequent occlusions.

Furthermore, while several existing systems require segmented or cropped images to improve performance, our ViT-based model processes full-frame images without any pre-processing. This design decision was motivated by the need to build a low-complexity and deployment-ready system that avoids the dependencies and fragility introduced by external segmentation or keypoint estimation libraries. Cropping and skeleton-based methods often suffer from failure modes in occluded or cluttered scenes, which are common in industrial environments. This design choice enhances deployment feasibility, allowing real-time inference directly on raw camera feeds, and this preserves important contextual cues from the environment. Such robustness is particularly relevant in industrial settings characterized by complex lighting, background clutter, and unpredictable occlusions.

Importantly, the SPECTRE-ViT system was explicitly designed for low-cost, scalable deployment in real-world industrial environments, including small- and medium-sized enterprises. Thanks to its compact architecture (∼5M parameters) and independence from external pre-processing (e.g., skeleton detection or image cropping), the model can perform real-time inference even on edge devices. Crucially, the model has been tested on raw RGB frames without relying on dedicated cameras or depth sensors. This makes it suitable for use with embedded cameras found in off-the-shelf smartphones or tablets. In combination with lightweight edge computing frameworks, SPECTRE-ViT can be deployed directly on mobile devices positioned in the workplace, enabling non-intrusive, cost-effective ergonomic monitoring without cloud connectivity or specialized hardware.

Collectively, these features underscore that our ViT-based system not only surpasses both IMU and prior CNN-based approaches but also advances the state of the art among vision-based ergonomic monitoring tools. It offers a compelling combination of classification accuracy, computational efficiency, and operational simplicity, making it suitable for scalable, real-time deployment in dynamic manufacturing environments. The ViT-based vision system offers a non-invasive, low-cost, and scalable alternative, suited to dynamic environments with varying lighting, perspectives, and occlusion patterns.

Compared to other posture monitoring systems, including our previous SPECTRE framework, the proposed approach yields superior results across all evaluated metrics, especially for anatomically challenging regions such as wrists. Table 5 provides a detailed comparison between the two architectures, highlighting improvements in accuracy, model simplicity, and hardware requirements. In addition, Table 6 positions our method relative to other recent literature in terms of input type, accuracy, and robustness. Most prior approaches rely on either joint estimation pipelines or handcrafted rule-based scoring, both of which introduce fragility under occlusion and impose computational burdens. Our method avoids these steps entirely and achieves higher generalization using a simpler end-to-end strategy.

Table 5 highlights the key improvements of the SPECTRE-ViT system over our previously published SPECTRE architecture. In particular, the ViT-based model achieves significantly better accuracy for difficult joints (e.g., Right Wrist), eliminates the need for image segmentation or skeleton reconstruction and supports real-time inference, even on embedded devices. These enhancements make it more suitable for scalable deployment in real-world industrial workflows. From a broader perspective, this work contributes to addressing the following three persistent challenges in the field: (1) occlusion resilience—mitigated through self-attention and full-frame processing; (2) dataset realism—enabled by using an industrially representative, multi-modal dataset; and (3) outcome linkage—by leveraging direct ergonomic labeling based on ground-truth motion capture data in alignment with the RULA methodology.

Despite the excellent results, some limitations should be acknowledged. First, the dataset is derived from a controlled reproduction of industrial tasks, which, although realistic, may not encompass the full range of variability in real production settings. Field validation in operational environments with real-time constraints and multiple operators is currently in progress and will be a critical next step to confirm the generalizability of the model under uncontrolled lighting, occlusion, and workflow conditions. Second, while the model operates in near real time, latency benchmarks under deployment scenarios are yet to be rigorously quantified. Lastly, expanding the classification taxonomy to cover additional ergonomic indices (e.g., REBA, OWAS) and complex postural interactions (e.g., multi-joint dynamics) could further enhance its applicability.

## 5. Conclusions

This study has presented a novel and efficient vision-based system for ergonomic risk assessment, built upon a lightweight ViT architecture. The proposed method leverages full-frame RGB images to predict posture-level ergonomic compliance for key anatomical regions without requiring pre-processing steps such as cropping, background removal, or skeleton tracking. The integration of this streamlined pipeline with a high-performing classification backbone resulted in consistently excellent metrics, with average F1-scores and AUC values exceeding 0.99 across all classes.

Compared to conventional CNN-based models and previously published approaches such as the SPECTRE framework, the ViT model proposed here offers notable advantages in terms of computational efficiency, classification accuracy, and adaptability to real-world conditions. This positions the model as a highly promising candidate for non-invasive, real-time ergonomic monitoring in industrial settings, especially in collaborative workspaces where wearable sensors may be intrusive or impractical.

Beyond technical performance, the methodological design prioritizes deployment feasibility. By removing the dependency on skeleton estimation or joint angle computation and reducing reliance on expensive hardware setups or expert calibration, the system becomes inherently more accessible to small and medium-sized enterprises (SMEs). This democratization of ergonomic assessment technology holds the potential to significantly impact workplace health and safety, particularly in sectors characterized by manual labor and high WMSD prevalence.

Moreover, the proposed architecture offers scalability and flexibility. The low parameter count (∼5M) ensures that it can be deployed on edge devices or embedded systems without requiring cloud-based processing. This opens the door to real-time ergonomic feedback loops, which could provide workers and supervisors with continuous, actionable insights during task execution—a key feature for proactive injury prevention and adaptive human–machine collaboration.

Nevertheless, the conclusions drawn must be contextualized within the limitations of the present study. While the dataset was acquired under realistic conditions and using dual-modality ground truth (RGB + IMU), it still represents a controlled laboratory environment. Future work should extend the validation phase to fully operational industrial floors, incorporating variations in lighting, background clutter, occlusion severity, and worker apparel. Furthermore, longitudinal studies assessing the long-term impact of deploying such systems on ergonomic outcomes and productivity are warranted.

From a technical standpoint, future research will explore the integration of temporal attention mechanisms to enable continuous pose tracking and risk accumulation over time. Additionally, expanding the posture taxonomy to include lower-limb assessments and dynamic task phases could further enhance coverage. Efforts to interface with robotic task allocation systems—as hinted by recent studies on ergonomic human–robot collaboration—are also under consideration.

In conclusion, the proposed ViT-based framework represents a significant step forward in the development of scalable, high-performance, and user-friendly ergonomic assessment tools. It successfully bridges the gap between laboratory-grade accuracy and field-level usability, paving the way for more intelligent, responsive, and inclusive safety management systems within Industry 5.0 paradigms. By enabling the early detection of risk factors and facilitating data-driven interventions, such systems contribute not only to occupational health but also to a more sustainable and human-centered model of industrial innovation.

## Figures and Tables

**Table 1:** Hyperparameter grid used for training the Vision Transformer.

Hyperparameter	Tested Values
batch_size	8, 16, 32, 64
optimizer	Adam, AdamW, Adamax
learning rate (LR)	$1\times 10^{-2}$, $1\times 10^{-3}$, $1\times 10^{-4}$
dropout	0.1, 0.3, 0.5
Patch size	8, 16, 32, 64
Embedding dim.	192, 256, 512, 1024
Depth	1, 2, 4, 6
qkv_bias	True or False

**Table 2:** Encoding of labels in results.

Labels	Coded Labels
Neck	NECK
Trunk	TRUNK
**R**ight **U**pper **A**rm	RUA
**R**ight **L**ower **A**rm	RLA
**R**ight **W**rist	RW
**L**eft **U**pper **A**rm	LUA
**L**eft **L**ower **A**rm	LLA
**L**eft **W**rist	LW

**Table 3:** Average results for the 5 folds for each class.

Labels	AUC	Accuracy	F1	Precision	Recall
NECK	0.9998	0.9961	0.9971	0.9971	0.9971
TRUNK	0.9999	0.9965	0.9974	0.9970	0.9977
RUA	0.9996	0.9928	0.9947	0.9962	0.9933
RLA	0.9986	0.9894	0.9930	0.9951	0.9909
RW	0.9962	0.9878	0.9927	0.9891	0.9963
LUA	0.9995	0.9944	0.9942	0.9947	0.9938
LLA	0.9999	0.9944	0.9964	0.9945	0.9983
LW	0.9995	0.9909	0.9932	0.9961	0.9904

**Table 4:** Results obtained in terms of Precision–Recall (PR-AUC) between the SPECTRE-ViT architecture and the previous SPECTRE architecture.

Labels	SPECTRE-ViT	SPECTRE [52]
NECK	**1.0000**	0.9870
TRUNK	**1.0000**	0.9950
RUA	**0.9997**	0.9600
RLA	**0.9990**	0.9230
RW	**0.9997**	0.8700
LUA	**0.9998**	0.9980
LLA	**0.9998**	0.9750
LW	**0.9986**	0.9700

Best values are highlighted in bold.

**Table 5:** Comparison between the original SPECTRE framework (2022) and the proposed SPECTRE-ViT model (2025).

	SPECTRE [52]	SPECTRE-ViT
Architecture	Parallel CNNs (one per body part)	Unified Vision Transformer (ViT) with multi-label output
Input Type	Segmented RGB + skeleton overlay	Raw RGB frames (full frame)
Preprocessing	Mediapipe segmentation + joint alignment	None (no cropping or skeleton extraction)
Number of Models	8 CNNs (independent classifiers)	Single ViT model (shared encoder)
Explainability	LIME applied to each CNN	Not applied (future work)
Lowest F1-score (Right Wrist)	0.9260	0.9927
Highest F1-score (Trunk)	0.9409	0.9974
Highest F1-score (Neck)	0.9362	0.9971
Inference Mode	Offline only	Near real-time
Hardware Requirements	High (A100 GPU)	Edge-capable (GTX-class or mobile)

**Table 6:** Comparison of SPECTRE-ViT with selected vision-based ergonomic monitoring systems.

Study	Input Type	Pipeline	Evaluation Metrics	Limitations
Nayak & Kim (2021) [36]	2D Joint Data (from images)	CNN-based joint localization → RULA scoring	ICC = 0.776–0.867 (reliability vs. experts)	No F1 reported; requires side-view image pair; dependent on person detection
Zhu et al. (2019) [35]	RGB video	Spatiotemporal CNN with spatial fusion	95–97% (action recognition)	Not task-agnostic; no ergonomic score estimation
Andrade-Ambriz et al. (2022) [34]	RGB video	Temporal CNN trained on short clips	Accuracy = 96.4% (human activity)	Not validated for ergonomic classification or real-time deployment
Aghamohammadi et al. (2024) [47]	RGB video (4-frame sequence)	Multi-route CNN with temporal fusion	F1 = 0.88	Temporal input required; occlusion robustness limited to short intervals
**SPECTRE-ViT (2025)**	Raw RGB (single frame)	Vision Transformer (ViT), direct multi-region classification	F1 > 0.99 (all regions)	Latency not evaluated; field tests ongoing

## Data Availability

Data are available upon reasonable request from the corresponding author.
